# Granulomatosis With Polyangiitis Presenting as a Rapidly Enlarging Choroidal Mass: A Case Report

**DOI:** 10.7759/cureus.95548

**Published:** 2025-10-27

**Authors:** Kosuke Aonuma, Munenari Kondo, Maki Suzuki, Hiroki Kaneko

**Affiliations:** 1 Department of Ophthalmology, Hamamatsu University School of Medicine, Hamamatsu, JPN

**Keywords:** antineutrophil cytoplasmic antibody (anca), choroidal mass, corticosteroid therapy, granulomatosis with polyangiitis (gpa), intraocular tumor, mpo-anca, ocular manifestation, scleritis

## Abstract

Granulomatosis with polyangiitis (GPA) is a necrotizing vasculitis that can involve virtually any organ system. Among ocular manifestations, choroidal involvement is exceedingly rare and may mimic intraocular tumors.

We report the case of a 68-year-old woman who presented with floaters and a rapidly enlarging choroidal mass. Imaging with contrast-enhanced CT revealed a choroidal lesion, tracheal wall thickening, pulmonary nodules, and maxillary sinus mucosal hypertrophy. FDG-PET demonstrated uptake in these lesions and in the nasal septum. Biopsies of the nasal septum and bronchus showed plasma cell and lymphocyte infiltration with vasculitic changes, providing histopathologic confirmation of vasculitis. Laboratory testing demonstrated elevated CRP and MPO-ANCA positivity. After excluding malignancy, infection, drug-induced vasculitis, and secondary vasculitides, a diagnosis of GPA was made. Oral prednisone (50 mg/day) was initiated, resulting in rapid regression of the choroidal lesion and normalization of MPO-ANCA levels.

This case highlights a rare ocular manifestation of GPA presenting as a rapidly enlarging choroidal mass. Awareness of such atypical presentations is crucial to avoid misdiagnosis as intraocular malignancy. Early systemic evaluation and prompt immunosuppressive therapy can lead to rapid resolution and favorable outcomes.

## Introduction

Granulomatosis with polyangiitis (GPA), formerly known as Wegener’s granulomatosis, is a necrotizing vasculitis that predominantly affects small- to medium-sized vessels. According to the 2012 Revised International Chapel Hill Consensus Conference (CHCC) Nomenclature of Vasculitides, GPA is categorized as one of the anti-neutrophil cytoplasmic antibody (ANCA)-associated vasculitides, characterized by necrotizing granulomatous inflammation and small-vessel vasculitis [1]. The disease typically involves the upper and lower respiratory tract and kidneys, but virtually any organ system may be affected [2].

The ocular manifestations of GPA include scleritis, peripheral ulcerative keratitis, orbital inflammation, nasolacrimal duct obstruction, and, less frequently, posterior segment lesions such as retinal or choroidal involvement [3]. The development of a choroidal tumor caused by GPA is rare, and it is important to consider GPA in the differential diagnosis of choroidal tumors [4].

In addition to ocular findings, GPA often involves disease confined to the upper or lower respiratory tract, reflecting its systemic vasculitic nature [1,2].

We report a rare case of GPA presenting as a rapidly enlarging choroidal mass that regressed dramatically following corticosteroid therapy. This case highlights the importance of considering systemic vasculitis in the differential diagnosis of atypical intraocular mass lesions. 

## Case presentation

**Figure 1:**
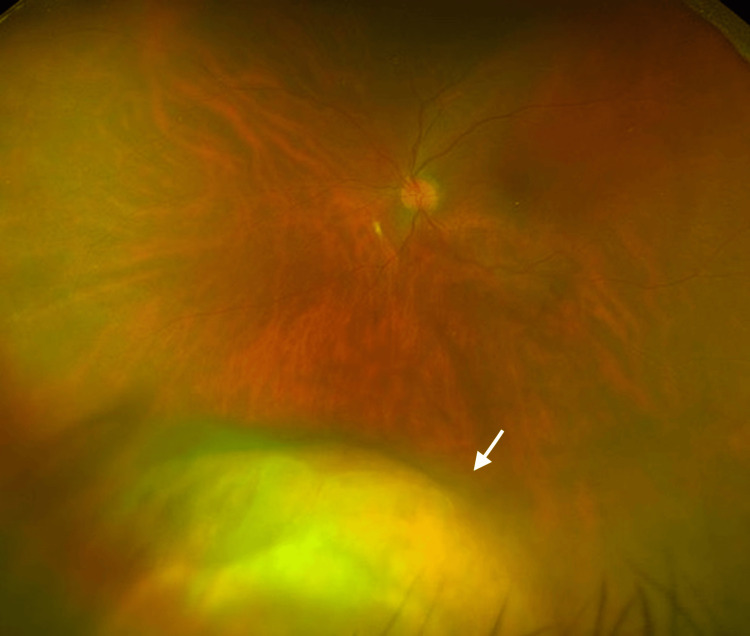
Fundus photograph showing a rapidly enlarging choroidal mass in the left eye (white arrow).

A 68-year-old woman presented with floaters and was found to have a left choroidal mass at another clinic. As the lesion demonstrated rapid enlargement, she was referred to our hospital (Figure 1). Her medical history included hypothyroidism, exudative otitis media, and hypertension. One year earlier, she had developed unexplained left iritis and scleritis, which had been controlled with topical corticosteroids. In the preceding several months, she had also experienced worsening hearing loss, dysgeusia, and loss of appetite.

**Figure 2:**
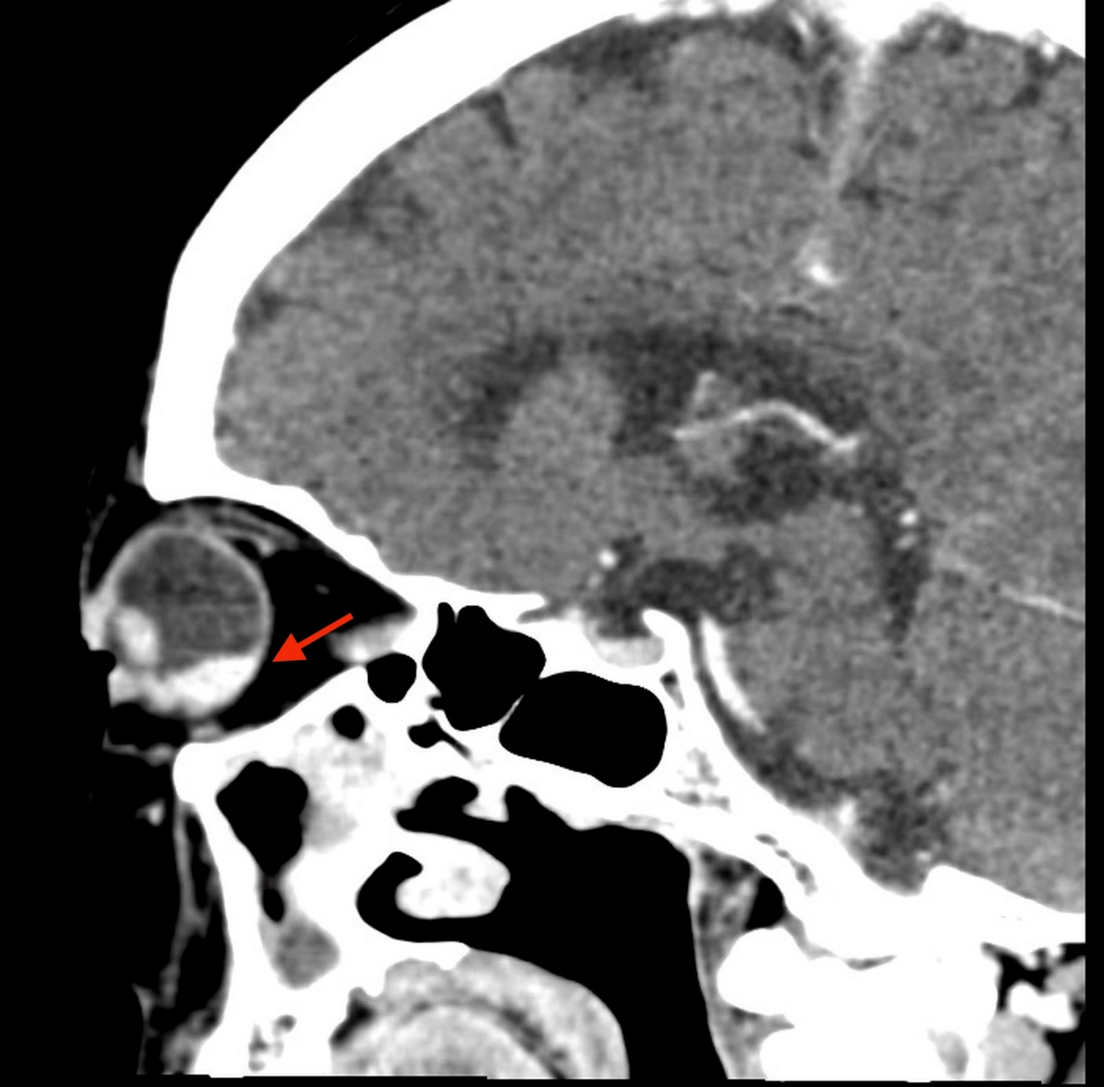
Contrast-enhanced CT showing a left choroidal lesion (red arrow).

**Figure 3:**
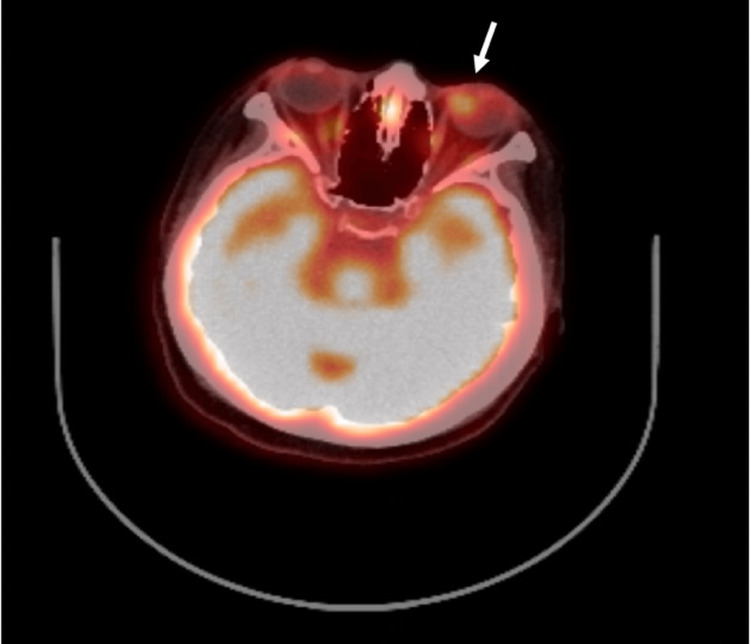
FDG-PET showing increased uptake in the left choroidal lesion (white arrow) and in the nasal septum.

On examination at our hospital, the best-corrected visual acuity was 20/30 in the right eye and 20/20 in the left eye, and fundus evaluation revealed a yellowish-white choroidal lesion with rapid growth, raising suspicion of a metastatic choroidal tumor. Accordingly, a systemic evaluation was performed. Contrast-enhanced CT demonstrated a left choroidal lesion, thickening extending from the cricoid cartilage to the tracheal wall, pulmonary nodules, and mucosal hypertrophy of the maxillary sinus, while no orbital abnormalities were observed (Figure 2). FDG-PET revealed increased uptake in these lesions as well as in the nasal septum (Figure 3). Biopsies of the nasal septum and bronchus showed infiltration of plasma cells and lymphocytes with vasculitic changes, without evidence of malignancy.

Laboratory testing demonstrated white blood cells (13,420 /μL), elevated C-reactive protein (CRP) (6.50 mg/dL), and MPO-ANCA positivity (243 U/mL) (Table 1). Blood cultures and β-D-glucan were negative. Urinalysis revealed no proteinuria, ruling out glomerulonephritis, and cerebrospinal fluid analysis showed no abnormalities, including protein elevation. After excluding malignancy, infection, drug-induced vasculitis, and secondary vasculitides, and with histologic confirmation of vasculitis in conjunction with the clinical findings, a diagnosis of GPA was established. MRI was considered to assess possible hypertrophic pachymeningitis, but could not be performed due to claustrophobia.

**Table 1:** Laboratory findings at presentation. White blood cells count and C-reactive protein (CRP) levels were elevated, and MPO-ANCA was positive.

Test	Reference range	Results
White blood cells	3,300–8,600 /μL	13,420
C-reactive protein (CRP)	<0.14 mg/dL	6.5
MPO-ANCA	<3.5 U/mL	243
PR3-ANCA	<3.5 U/mL	<1.0
β-D-glucan	Negative	Negative
Blood culture	Negative	Negative
Urinalysis (protein)	Negative	Negative
Cerebrospinal fluid protein	10-40 mg/dL	33

**Figure 4:**
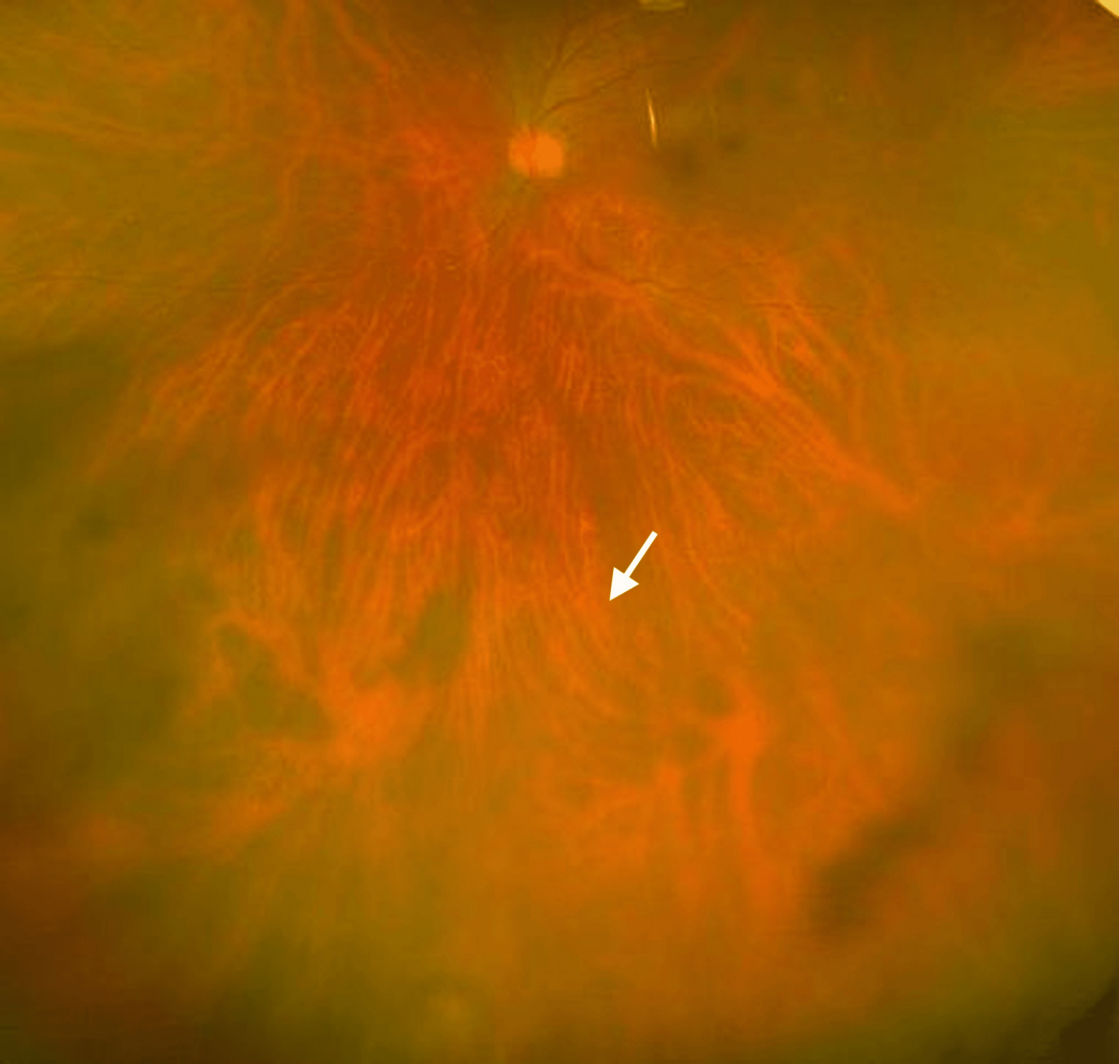
Regression of the choroidal lesion after corticosteroid therapy (white arrow indicates the same area as in Figure 1).

**Figure 5:**
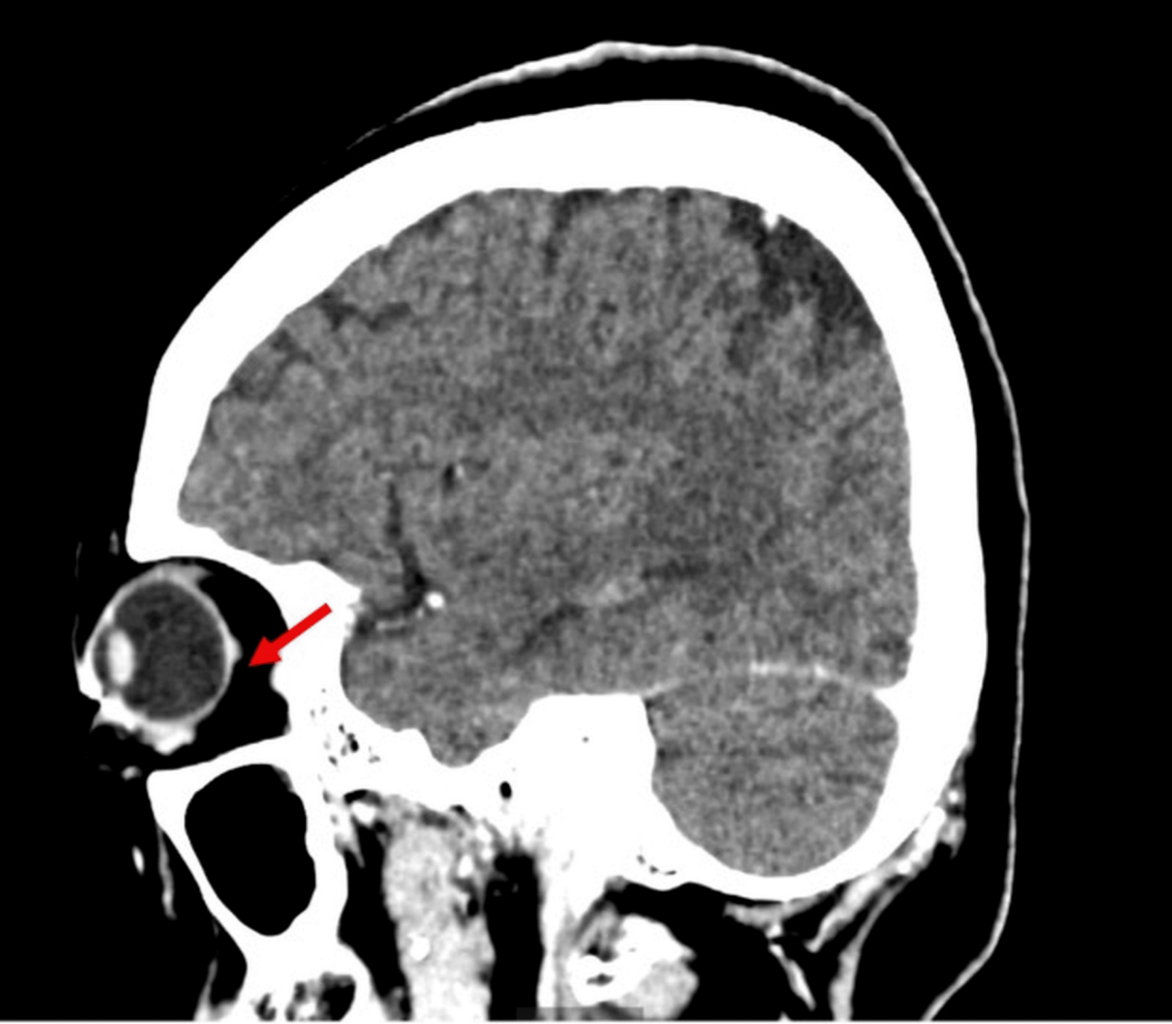
Follow-up CT showing regression of the left choroidal lesion (red arrow indicates the same area as in Figure 2).

Oral prednisone (50 mg/day, approximately 1.0 mg/kg/day based on a body weight of 52 kg) was initiated, and the choroidal lesion rapidly regressed within several days (Figure 4). A follow-up CT performed nine days after starting corticosteroid therapy showed marked regression of the choroidal mass, and the patient has since remained stable under gradual tapering (Figure 5). Laboratory findings showed decreases in white blood cells (7,850 /μL), C-reactive protein (CRP) (0.02 mg/dL), and MPO-ANCA (2.5 U/mL) (Table 2). Prednisone was gradually tapered, and the patient remained recurrence-free during follow-up. The dramatic response of the choroidal lesion to corticosteroid therapy strongly suggested that it was attributable to GPA.

**Table 2:** Laboratory findings after corticosteroid therapy. White blood cells count, C-reactive protein (CRP), and MPO-ANCA levels all showed improvement.

Test	Reference range	Results
White blood cells	3,300–8,600 /μL	7850
C-reactive protein (CRP)	<0.14 mg/dL	0.02
MPO-ANCA	<3.5 U/mL	2.5

## Discussion

GPA is exceedingly rare but clinically important, as it may mimic intraocular tumors. The present case showed a rapidly enlarging choroidal mass that regressed dramatically with corticosteroid therapy, underscoring the potential for inflammatory choroidal lesions to represent a manifestation of GPA and their responsiveness to immunosuppression.

Ophthalmic manifestations are well recognized in GPA, including orbital disease, abnormalities of the sclera and cornea, and nasolacrimal duct obstruction [3]. Rothschild et al. analyzed 1,286 patients with systemic necrotizing vasculitides and found ophthalmologic involvement in about 16.6% at the time of diagnosis, with GPA being the leading cause among these entities [5]. These data emphasize both the diversity of ocular disease in GPA and the exceptional rarity of direct choroidal involvement, such as observed in our patient.

Previous literature has reported only a handful of similar cases. Masuda et al. described an 83-year-old woman with a choroidal tumor due to GPA, where histopathology revealed necrotizing vasculitis and granulomatous inflammation, and treatment with corticosteroid plus azathioprine induced rapid regression [4]. Similarly, Proia reported granulomatous choroiditis in Wegener’s granulomatosis, highlighting that direct granulomatous inflammation of the choroid, though extremely rare, belongs to the ocular spectrum of GPA [6].

Choroidal involvement in GPA results from inflammatory occlusion of the choriocapillaris, leading to ischemia and infarction of the choriocapillaris-RPE-outer retina complex. In advanced stages, granulomatous inflammation infiltrates the choroid, causing stenosis or occlusion of the capillaries and dense inflammatory infiltration, consistent with granulomatous choroiditis [6-8]. Understanding this mechanism helps explain how choroidal lesions in GPA can appear as mass-like lesions yet respond dramatically to corticosteroid therapy.

Although PR3-ANCA positivity is more typically associated with GPA in Western populations, several studies from Asia have reported a higher prevalence of MPO-ANCA-positive GPA. In a cohort of Chinese patients, Chen et al. demonstrated that over 60% of those diagnosed with GPA were MPO-ANCA positive, showing a tendency toward more frequent renal involvement and less frequent ear or ocular disease compared with PR3-ANCA-positive cases [9]. These findings indicate that MPO-ANCA-associated GPA represents a distinct clinical phenotype, which may partially explain the presentation observed in our patient.

Similarly, a Japanese case series by Matsuo reported that patients with MPO-ANCA-associated vasculitis frequently develop ocular manifestations such as scleritis, peripheral keratitis, retinal vein occlusion, and optic neuropathy [10]. These observations suggest that although ocular involvement is less common in MPO-ANCA-positive GPA than in PR3-ANCA-positive disease overall, ocular vasculitic complications can still occur and may present with variable severity and morphology across populations.

Scleritis is a frequent ocular manifestation of GPA, and GPA-associated scleritis tends to be more severe than that associated with other etiologies [11]. In addition, Demirci et al. described a giant nodular posterior scleritis initially mistaken for choroidal melanoma, but confirmed as inflammatory by biopsy, underscoring how posterior scleritis can closely simulate intraocular tumors [12].

Taken together, these reports and our case indicate that GPA can manifest with diverse and atypical ocular lesions ranging from common scleritis to, in rare instances, choroidal masses. Clinicians should maintain a high index of suspicion for GPA in patients presenting with unusual intraocular or orbital mass-like lesions, and early systemic evaluation and prompt immunosuppressive therapy are essential to improve outcomes.

## Conclusions

We report a rare case of GPA presenting as a choroidal mass. Awareness of this atypical ocular manifestation is crucial to avoid misdiagnosis as malignancy. In patients with atypical intraocular mass-like lesions, GPA should be considered in the differential diagnosis, and systemic evaluation is warranted to identify possible multisystem involvement. Early initiation of immunosuppressive therapy can lead to rapid ocular recovery and may also prevent potentially life-threatening systemic complications.

## References

[1] Jennette JC, Falk RJ, Bacon PA (2013). 2012 revised International Chapel Hill Consensus Conference Nomenclature of Vasculitides. Arthritis Rheum.

[2] Holle JU, Gross WL, Holl-Ulrich K (2010). Prospective long-term follow-up of patients with localised Wegener's granulomatosis: does it occur as persistent disease stage?. Ann Rheum Dis.

[3] Bullen CL, Liesegang TJ, McDonald TJ, DeRemee RA (1983). Ocular complications of Wegener's granulomatosis. Ophthalmology.

[4] Masuda T, Izumi Y, Takeshita H (2015). Granulomatosis with polyangiitis presenting as a choroidal tumor. Case Rep Rheumatol.

[5] Rothschild PR, Pagnoux C, Seror R, Brézin AP, Delair E, Guillevin L (2013). Ophthalmologic manifestations of systemic necrotizing vasculitides at diagnosis: a retrospective study of 1286 patients and review of the literature. Semin Arthritis Rheum.

[6] Proia AD (2011). Granulomatous choroiditis in Wegener granulomatosis. Arch Ophthalmol.

[7] Chiquet C, Lumbroso L, Denis P, Papo T, Durieu I, Lehoang P (1999). Acute posterior multifocal placoid pigment epitheliopathy associated with Wegener's granulomatosis. Retina.

[8] Özdal PÇ, Tugal-Tutkun I (2022). Choroidal involvement in systemic vasculitis: a systematic review. J Ophthalmic Inflamm Infect.

[9] Chen M, Yu F, Zhang Y, Zou WZ, Zhao MH, Wang HY (2005). Characteristics of Chinese patients with Wegener's granulomatosis with anti-myeloperoxidase autoantibodies. Kidney Int.

[10] Matsuo T (2007). Eye manifestations in patients with perinuclear antineutrophil cytoplasmic antibody-associated vasculitis: case series and literature review. Jpn J Ophthalmol.

[11] Sainz de la Maza M, Foster CS, Jabbur NS (1995). Scleritis associated with systemic vasculitic diseases. Ophthalmology.

[12] Demirci H, Shields CL, Honavar SG, Shields JA, Bardenstein DS (2000). Long-term follow-up of giant nodular posterior scleritis simulating choroidal melanoma. Arch Ophthalmol.

